# HMGB1 released from intestinal epithelia damaged by cholera toxin adjuvant contributes to activation of mucosal dendritic cells and induction of intestinal cytotoxic T lymphocytes and IgA

**DOI:** 10.1038/s41419-018-0665-z

**Published:** 2018-05-24

**Authors:** Ayako Wakabayashi, Masumi Shimizu, Eiji Shinya, Hidemi Takahashi

**Affiliations:** Department of Microbiology and Immunology, Nippon Medical School, 1-1-5 Sendagi, Bunkyo-ku, Tokyo, 113-8602 Japan

## Abstract

Cholera toxin (CT) is a potent mucosal adjuvant and oral administration of ovalbumin (OVA) antigens plus CT induces OVA-specific CD8^+^ cytotoxic T lymphocytes (CTLs) and IgA production in intestinal mucosa. However, the mechanisms of induction of these immune responses remain unknown. Intestinal OVA-specific CD8^+^ CTLs were not induced by oral administration of the CT active (CTA) or CT binding (CTB) subunit as an adjuvant and CD11c^+^ DCs were involved in cross-priming of intestinal CTLs. CD8^+^CD103^+^CD11c^+^CD11b^−^DCs and DCIR2^+^CD103^+^CD11c^+^CD11b^+^ DCs were distributed in the intestinal lamina propria and mesenteric lymph nodes, both DC subsets expressed DEC-205, and the expression of co-stimulatory molecules such as CD80 and CD86 was enhanced in both DC subsets after oral administration of intact CT but not the CTA or CTB subunit. Intestinal DCs activated by the oral administration of OVA plus CT cross-presented OVA antigens and DCs that captured OVA antigen through DEC-205, but not DCIR2, could cross-present antigen. We found that oral administration of intact CT, but not the CTA or CTB subunit, enhanced cell death, cytoplasmic expression of high-mobility group box 1 protein (HMGB1) in epithelial cell adhesion molecule (EpCAM)^+^CD45^−^ intestinal epithelial cells (IECs), and HMGB1 levels in fecal extracts. HMGB1 dose-dependently enhanced the expression of CD80 and CD86 on DCs in vitro, and intravenous or oral administration of glycyrrhizin, an HMGB1 inhibitor, significantly suppressed activation of mucosal DCs and induction of intestinal OVA-specific CTLs and IgA by oral CT administration. These results showed that oral administration of intact CT triggers epithelial cell death in the gut and the release of HMGB1 from damaged IECs, and that the released HMGB1 may mediate activation of mucosal DCs and induction of CTLs and IgA in the intestine.

## Introduction

Cholera toxin (CT) is a potent mucosal adjuvant and oral administration of an antigen plus CT induces antigen-specific mucosal IgA and plasma IgG production^1^. We previously reported that oral administration of ovalbumin (OVA) plus CT adjuvant predominantly induces OVA-specific cytotoxic T lymphocytes (CTLs) in gastrointestinal intraepithelial lymphocytes (IELs) and successfully suppresses growth of OVA-expressing tumor implanted in C57BL/6 (B6) mice^2^.

In some situations, CTL epitopes within exogenous protein antigens are presented on major histocompatibility complex (MHC) class I professional antigen-presenting cells, such as dendritic cells (DCs), to naive CD8^+^ T cells^3–5^. This phenomenon is called “cross-presentation” and is exhibited by CD8^+^ DCs^6^ and CD103^+^ DCs^7^. Effective induction of exogenous antigen cross-presentation by DCs and subsequent priming of CTLs is important in vaccine development for tumors and pathogens. CD103^+^CD8α^+^ DCs that are CD11c^hi^CD11b^lo^ subsets in the intestinal lamina propria (LP) have been shown to induce CTL activity in vivo^8^. Moreover, DEC-205^+^ DC subset and DCIR2^+^ DC subset have been shown to be associated with cross-presentation via MHC class I and presentation by MHC class II, respectively^9^. Both DEC-205 and DCIR2 belong to the C-type lectin family, which is involved in the capture, endocytosis, and processing of glycoprotein antigen^10^.

CT from *Vibrio cholerae* comprises one toxic A subunit with ADP-ribosyltransferase activity and five nontoxic B-subunits that are responsible for binding to monosialoganglioside 1 on the cell surface^11,12^. We previously showed that unlike oral CT administration, oral administration of the CT binding (CTB) subunit cannot induce antigen-specific CTLs and suppress tumor growth^2^. Therefore, we investigated how oral CT adjuvant induces antigen-specific CTLs in intestinal tissues and why the CT active (CTA) or CTB subunit cannot prime these CTLs.

Intact CT has been shown to accelerate cell death of epithelial cells from rabbit ileum^13^ and trigger apoptosis in human cell lines^14^ and a murine cell line^15^. Dying, damaged, or stressed cells extracellularly release damage-associated molecular pattern (DAMP) molecules, such as high-mobility group box 1 protein (HMGB1), which is a non-histone nuclear protein, and the released DAMP molecules cause inflammation^16,17^. HMGB1 acts as an activator of DCs and upregulates the expression of co-stimulatory molecules, including CD80 and CD86, on human DCs^18^ and rat DCs^19^.

In the present study, we assessed the expression of DEC-205 on intestinal CD103^+^CD11b^−^ DCs and CD103^+^CD11b^+^ DCs^20^. Moreover, we examined whether co-stimulatory molecules that were enhanced on each DC subset and these DCs could cross-present antigen by oral administration of intact CT, the CTA subunit, or the CTB subunit. Finally, we examined whether the intestinal epithelial cell (IEC) damage and HMGB1 release were enhanced by oral CT, CTA, or CTB, and whether HMGB1 mediated DC activation, cross-presentation of antigen, and Ig production.

## Results

### Expression of DEC-205 on both CD8^+^CD103^+^CD11b^−^ DCs and CD103^+^CD11b^+^ DCs in the intestinal LP and mesenteric lymph nodes

**Fig. 1:**
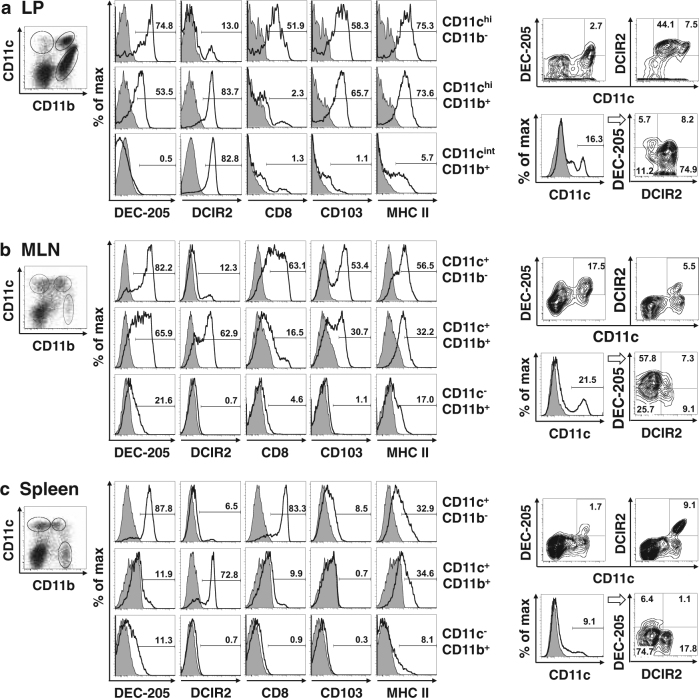
Expression of DEC-205 on both CD8^+^CD11b^−^ DCs and DCIR2^+^CD11b^+^ DCs in intestinal LP and MLNs. **a** Intestinal LP cells, **b** MLN cells, and **c** splenic cells were isolated from B6 mice, triple-stained with FITC-labeled anti-CD11b, APC-labeled anti-CD11c, and PE-labeled anti-DEC-205, anti-DCIR2, anti-CD8, anti-CD103, anti-MHC class II Ab, or subtype control Ab (gray fill), and analyzed by flow cytometry. CD11c^hi/+^CD11b^−^, CD11c ^hi/+^CD11b^+^, and CD11c^int/-^CD11b^+^ cells were gated, and the expression of DEC-205, DCIR2, CD8, CD103, or MHC class II (bold solid line) was analyzed. The cells were also triple-stained with FITC-labeled anti-CD11c, APC-labeled anti-DCIR2, and PE-labeled anti-DEC-205 Ab. CD11c^hi/+^ DCs were gated and the expression of DEC-205 and DCIR2 was analyzed. Each value represents the percentage of cells expressing each molecule. Data are representative of two independent experiments

Initially, we assessed the distribution of CD8^+^CD103^+^CD11b^−^ DCs and CD103^+^CD11b^+^ DCs^20^ in the intestinal LP, mesenteric lymph nodes (MLNs), and the spleen from mice, and analyzed the expression of DEC-205 or DCIR2 on DCs. CD11c^hi/+^CD11b^−^ DCs that were positive for CD8, CD103, and MHC class II in the LP and MLNs clearly expressed DEC-205 but not DCIR2 (Fig. 1a,b). CD11c^hi/+^CD11b^+^ DCs that were positive for CD103 and MHC class II in the LP and MLNs strongly expressed both DEC-205 and DCIR2 (Fig. 1a,b). In the spleen, DEC-205 was expressed only on CD11c^+^CD11b^−^ DCs that were positive for CD8, CD103, and MHC class II, whereas DCIR2 was expressed only on CD11c^+^CD11b^+^MHCII^+^ DCs (Fig. 1c). We also confirmed that DEC-205^+^DCIR2^+^ double-positive CD11c^hi/+^ cells were distributed in the LP and MLNs (Fig. 1a,b) but not in the spleen (Fig. 1c). CD11c^int^CD11b^+^ cells in the intestinal LP are eosinophils that express CD80^21^.

These results show that DEC-205 is expressed not only on CD8^+^CD103^+^CD11b^−^ DCs but also on DCIR2^+^CD103^+^CD11b^+^ DCs in the intestinal LP and MLNs, in contrast to the splenic expression of DEC-205 confined to CD8^+^CD103^+^CD11b^−^ DCs.

### Enhancement of CD80 and CD86 expression on both DEC-205^+^CD8^+^CD11b^−^ DCs and DEC-205^+^DCIR2^+^CD11b^+^ DCs in the intestinal LP and MLNs after oral CT administration

**Fig. 2:**
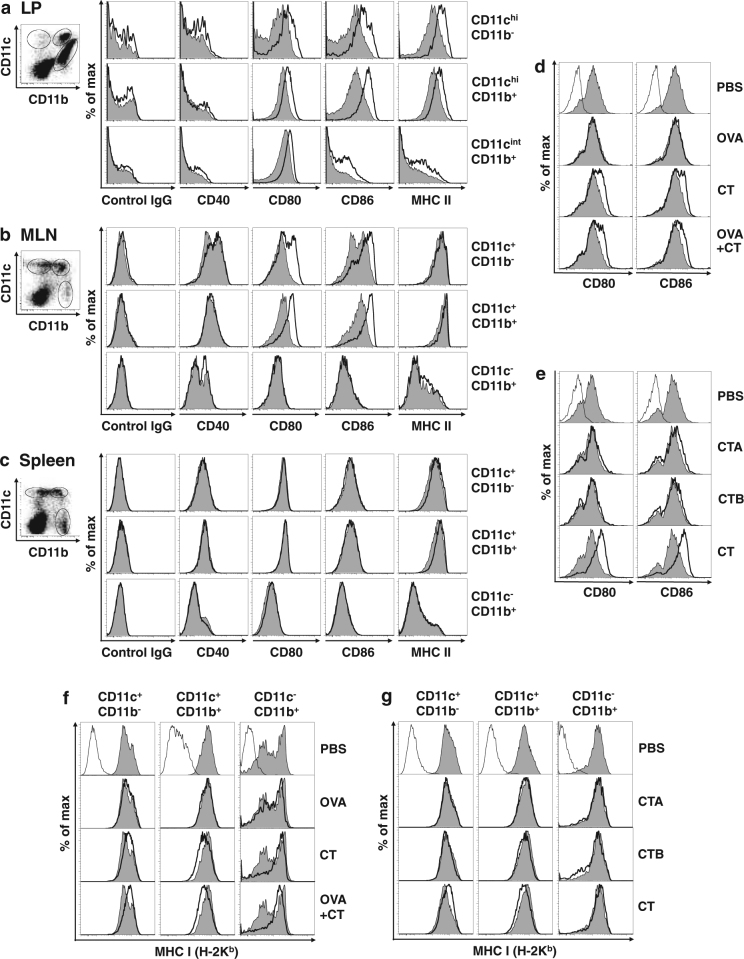
Enhancement of CD80 and CD86 expression on both DEC-205^+^CD8^+^CD11b^−^ DCs and DEC-205^+^DCIR2^+^CD11b^+^ DCs in intestinal LP and MLNs after oral administration of intact CT. **a**, **b**, **c** B6 mice were orally administered 10 μg of CT (bold solid line) or PBS (gray fill). Intestinal LP cells (**a**) were isolated from the mice at 16 h after oral administration, and MLN cells (**b**) and splenic cells (**c**) were isolated at 24 h. The cells were triple-stained with FITC-labeled anti-CD11b, APC-labeled anti-CD11c, and PE-labeled anti-CD40, anti-CD80, anti-CD8, anti-CD86, anti-MHC class II Ab, or subtype control Ab, and analyzed by flow cytometry. CD11c^hi/+^CD11b^−^, CD11c ^hi/+^CD11b^+^, and CD11c^int/−^CD11b^+^ cells were gated, and the expression of CD40, CD80, CD86, or MHC class II was analyzed. **d** B6 mice were orally administered PBS, 100 mg of OVA alone, 10 μg of CT alone, or OVA plus CT. MLN cells were isolated from the mice 24 h after oral administration and double-stained with FITC-labeled anti-CD11c and PE-labeled anti-CD80, anti-CD86 (gray fill for PBS-treated mice and bold solid line for mice treated with OVA, CT, or OVA plus CT), or subtype control Ab (solid line). CD11c^+^ cells were gated and the expression of CD80 or CD86 was analyzed. **e** B6 mice were orally administered 10 μg of CTA, CTB, or intact CT. MLN cells were isolated and the expression of CD80 or CD86 on CD11c^+^ cells was analyzed as described in (**d**). **f** B6 mice were orally administered PBS, 100 mg of OVA alone, 10 μg of CT alone, or OVA plus CT. MLN cells were isolated from the mice 24 h after oral administration and triple-stained with FITC-labeled anti-CD11b, APC-labeled anti-CD11c, and PE-labeled anti-MHC class I (H-2K^b^) Abs (gray fill for PBS-treated mice and bold solid line for mice treated with OVA, CT, or OVA plus CT) or subtype control Ab (solid line). CD11c^+^CD11b^−^, CD11c^+^CD11b^+^, and CD11c^−^CD11b^+^ cells were gated, and the expression of MHC class I was analyzed. **g** B6 mice were orally administered 10 μg of CTA, CTB, or intact CT. MLN cells were isolated and the expression of MHC class I on CD11c^+^CD11b^−^, CD11c^+^CD11b^+^, and CD11c^−^CD11b^+^ cells was analyzed as described in (**f**). Data are representative of two independent experiments

We analyzed whether expression of co-stimulatory molecules, such as CD40^22,23^, CD80, and CD86^24,25^, was enhanced on intestinal and splenic DCs after oral administration of CT adjuvant. Enhancement of CD80 and CD86 expression was strongest at 12–16 h on LP CD11c^hi^ DCs and at 16–24 h on MLN CD11c^+^ DCs after oral CT administration (Fig. S1). The expression levels of CD80, CD86, and MHC class II were substantially enhanced on both CD11c^hi^CD11b^−^ DCs and CD11c^hi^CD11b^+^ DCs in the intestinal LP 16 h after oral CT administration (Fig. 2a). In MLNs at 24 h, CD80 and CD86 levels were enhanced on both CD11c^+^CD11b^−^ DCs and CD11c^+^CD11b^+^ DCs, but MHC class II expression on CD11c^+^ DCs was already high in a steady state^26^, and therefore clearly not enhanced further (Fig. 2b). CD40 expression was not increased on any cells in the LP, MLNs, or spleen (Fig. 2 and Fig. S1). We also confirmed the strong enhancement of CD80 and CD86 on both DEC-205^+^CD11c^hi/+^ DCs and DCIR^+^CD11c^hi/+^ DCs in the LP and MLNs after oral CT administration (Fig. S2). Oral administration of OVA alone, the CTA subunit, or the CTB subunit could not activate LP DCs (data not shown) and MLN DCs (Fig. 2d,e). MHC class I (H-2K^b^) expression was upregulated on CD11c^+^CD11b^−^ and downregulated on CD11c^+^CD11b^+^ DCs in the MLNs by oral CT administration, but the phenomenon was not observed in the LP and spleen or by oral administration of OVA alone, the CTA subunit, or the CTB subunit (Fig. 2f,g).

These results clearly demonstrate that oral administration of intact CT enhances CD80 and CD86 levels on both DEC-205^+^CD8^+^CD11b^−^ DCs and DEC-205^+^DCIR2^+^CD11b^+^ DCs in the intestinal LP and MLNs, but the CTA or CTB subunit cannot activate these DCs.

### Requirement of CD11c^+^ DCs for in vivo cross-priming of antigen-specific CD8^+^ CTLs in IELs

**Fig. 3:**
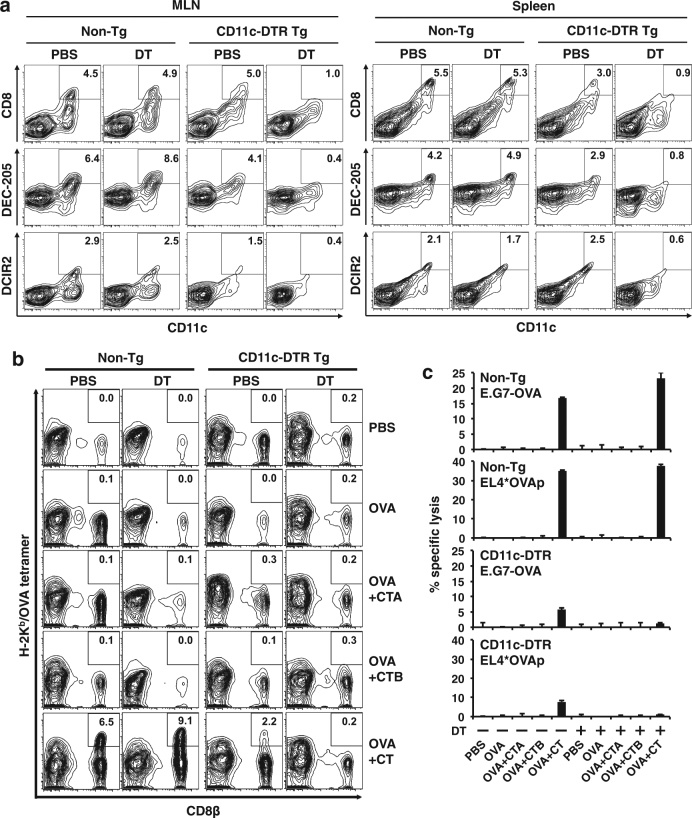
Intestinal OVA-specific CTLs cannot be induced in CD11c^+^ DC-depleted mice. **a** CD11c-DTR Tg mice and non-Tg littermates were i.p. injected with 4 ng of DT/g of body weight. One day later, MLN cells and SP cells were isolated from the mice, double-stained with FITC-labeled anti-CD11c Ab and PE-labeled anti-CD8, anti-DEC-205, or anti-DCIR2 Ab, and analyzed by flow cytometry. **b**, **c** CD11c-DTR Tg mice and non-Tg littermates were i.p. injected with PBS or DT on days 0 and 5, and orally administered 100 mg of OVA alone or plus 10 μg of CTA subunit, CTB subunit, or CT on days 1 and 6. IELs were isolated from the mice 2 days after the second oral administration, double-stained with PE-labeled H-2K^b^/OVA tetramer-SIINFEKL and APC-labeled anti-CD8β Ab, and analyzed by flow cytometry (**b**). OVA-specific CTL responses of the IELs as described in (**b**) were measured by the ^51^Cr-release assay using EL4 cells pulsed with 4 μM OVA-peptide SIINFEKL and E.G7-OVA cells as target cells (**c**). The effector:target (E:T) ratio was 50:1. Data are shown as the mean + SE in triplicate. The results are representative of two independent experiments

To determine whether CD11c^+^ DCs were essential for the cross-priming of CTLs, we used CD11c-DTR transgenic (Tg) mice^27^. CD11c^+^ DCs expressing CD8, DEC-205, or DCIR2 were clearly depleted in both the MLNs and spleen of CD11c-DTR Tg mice 1 day after intraperitoneal (i.p.) injection of diphtheria toxin (DT) and the DT-induced DC depletion persisted for 4 days (Fig. 3a). H-2K^b^ OVA tetramer-positive CD8β^+^ T cells and OVA-specific CTL activity were induced in IELs of DT- or phosphate-buffered saline (PBS)-injected non-Tg mice and PBS-injected CD11c-DTR Tg mice when the mice were orally administered OVA plus CT twice (Fig. 3b,c). However, in CD11c-DTR Tg mice depleted of CD11c^+^ DCs by i.p. injection of DT, tetramer-positive CD8^+^ T cells and CTL activity were not observed in the IELs, even when the same oral immunization was performed (Fig. 3b,c). Oral administration of OVA alone or with the CTA or CTB subunits did not induce tetramer-positive CD8^+^ T cells and CTL activity in any mice (Fig. 3b,c).

These results showed that CD11c^+^ DCs that express CD8, DEC-205, or DCIR2 are involved in cross-priming of intestinal antigen-specific CTLs by oral immunization with antigen plus CT.

### Intestinal DCs after the oral administration of OVA plus CT cross-present OVA antigens captured by the DEC-205 receptor

**Fig. 4:**
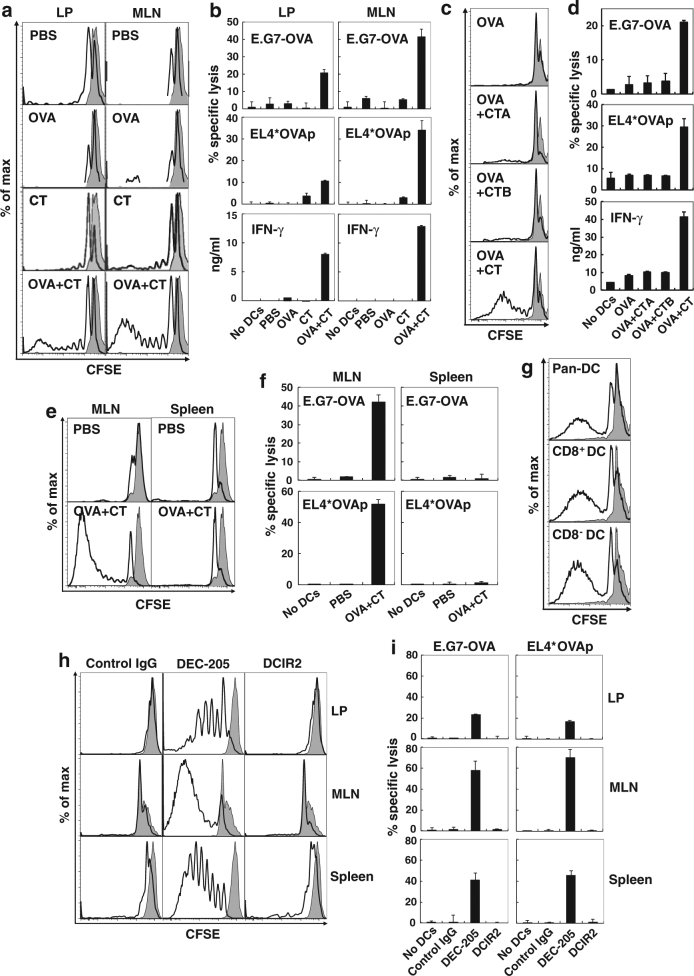
Intestinal DCs after oral administration of OVA plus CT cross-presenting OVA antigens captured by the DEC-205 receptor. **a** B6 mice were orally administered PBS, 10 mg of OVA alone, 10 μg of CT alone, or OVA plus CT. After 16 h, CD11c^+^ DCs were purified from LP and MLNs of the mice using the MACS system and co-cultured with CFSE-labeled OT-I T cells for 4 days. The cultured OT-I T cells were stained with APC-labeled anti-CD8 Ab. The CFSE-dilution profiles of CD8^+^ OT-I T cells were analyzed by flow cytometry. **b** The cultured OT-I T cells as described in (**a**) were re-stimulated by plate-bound anti-CD3 Ab for 16 h. OVA-specific cytotoxic activity of the re-stimulated OT-I T cells was measured by the ^51^Cr-release assay using EL4 cells pulsed with the OVA-peptide SIINFEKL and using E.G7-OVA cells as target cells. The E:T ratio was 10:1. IFN-γ release of the re-stimulated OT-I T cells was also assessed. The results are shown as the mean + SE in duplicate. **c**, **d** B6 mice were orally administered 10 mg of OVA alone or plus 10 μg of CTA subunit, CTB subunit or CT. After 24 h, CD11c^+^ DCs were purified from MLNs and co-cultured with CFSE-labeled OT-I T cells. The CFSE dilution profiles (**c**) and OVA-specific cytotoxic activity (**d**) were assessed as described in **a** and **b**, respectively. **e**, **f** B6 mice were orally administered PBS or 10 mg of OVA plus 10 μg of CT. After 24 h, CD11c^+^ DCs were purified from spleens and MLNs and co-cultured with OT-I T cells. After 4 days of culture, the CFSE dilution profiles (**e**) and OVA-specific cytotoxic activity (**f**) were assessed as described in **a** and **b**, respectively. **g** B6 mice were orally administered 10 mg of OVA plus 10 μg of CT. After 16 h, CD11c^+^ DCs, CD8^+^CD11c^+^ DCs, or CD8^−^CD11c^+^ DCs were purified from MLN cells using the MACS system and co-cultured with CFSE-labeled OT-I T cells for 4 days. The CFSE-dilution profiles were assessed as described in (**a**. **h**, **i**) CD11c^+^ DCs were purified from LP, MLNs, or spleens of B6 mice using the MACS system and labeled using biotinylated anti-DEC-205, anti-DCIR2, or subtype control Ab. Subsequently, the cells were loaded with anti-biotin Ab conjugated to OVA antigens and co-cultured with CFSE-labeled OT-I T cells for 4 days. The CFSE-dilution profiles (**h**) and OVA-specific cytotoxic activity **i** were assessed as described in **a** and **b**, respectively. The E:T ratio is 4:1 and the results are shown as the mean + SE in duplicate. The results are representative of two independent experiments

We further analyzed the cross-presentation ability of CD11c^+^ DCs in the intestinal LP, MLNs, or spleen of mice orally administered OVA plus CT. CD11c^+^ DCs were purified from intestinal LP and MLNs 16 h after oral administration of OVA alone, CT alone, or OVA plus CT and co-cultured with carboxyfluorescein succinimidyl ester-labeled OVA-specific OT-I T cells^28^. Four days later, CD11c^+^ DCs in the LP or MLNs from the mice that were orally administered OVA plus CT showed strong induction of CD8^+^ OT-I T-cell proliferation (Fig. 4a). Substantial OVA-specific cytotoxic activity and interferon (IFN)-γ release were also observed in OT-I T cells cultured with LP DCs or MLN DCs from mice that were orally administered OVA plus CT (Fig. 4b). CD11c^+^ DCs in the LP or MLNs from mice orally administered OVA alone, CT alone, or OVA plus CTA or CTB subunit did not induce proliferation and cytotoxic activity of OT-I T cells (Fig. 4a–d). Splenic DCs at 24 h (Fig. 4e,f), 3 days, or 5 days (data not shown) after oral immunization of OVA plus CT also did not induce OT-I T-cell proliferation and CTL responses.

Not only CD11c^+^ pan-DCs and CD8^+^CD11c^+^ DCs but also CD8^−^CD11c^+^ DCs purified from the MLNs of mice that were orally administered OVA plus CT could induce CD8^+^ OT-I T-cell proliferation (Fig. 4g). As both MLN CD8^+^ DCs and CD8^−^ DCs expressed DEC-205 (Fig. 1b), we examined whether the DEC-205 molecule on DCs was critical for the cross-presentation ability. To assess this possibility, we delivered OVA antigens to CD11c^+^ DCs from the LP, MLNs, or spleen through the DEC-205 or DCIR2 receptor. For effective antigen targeting, CD11c^+^ DCs were first labeled using biotinylated anti-DEC-205 antibody (Ab), anti-DCIR2 Ab, or subtype control Ab, and subsequently, the cells were loaded with anti-biotin Ab conjugated to OVA antigens. Accordingly, OVA antigen delivered through the DEC-205 receptor could be cross-presented by not only LP DCs and MLN DCs but also splenic DCs (Fig. 4h,i).

These results demonstrate that after oral administration of OVA plus CT, intestinal DCs that capture OVA antigen by the DEC-205 receptor may cross-present OVA antigen but splenic DCs were unable to cross-present OVA antigens in vivo, although DEC-205^+^ DCs from any tissues possess cross-presentation ability in vitro. This in vivo cross-presentation ability is probably due to the enhanced expression of CD80 and CD86 on DEC-205^+^ LP DCs or MLN DCs (Fig. 2, Fig. S2) but not on splenic DCs (Fig. 2c).

### Enhancement of epithelial cell death, cytoplasmic HMGB1 expression in IECs, and increased HMGB1 release by oral CT administration

**Fig. 5:**
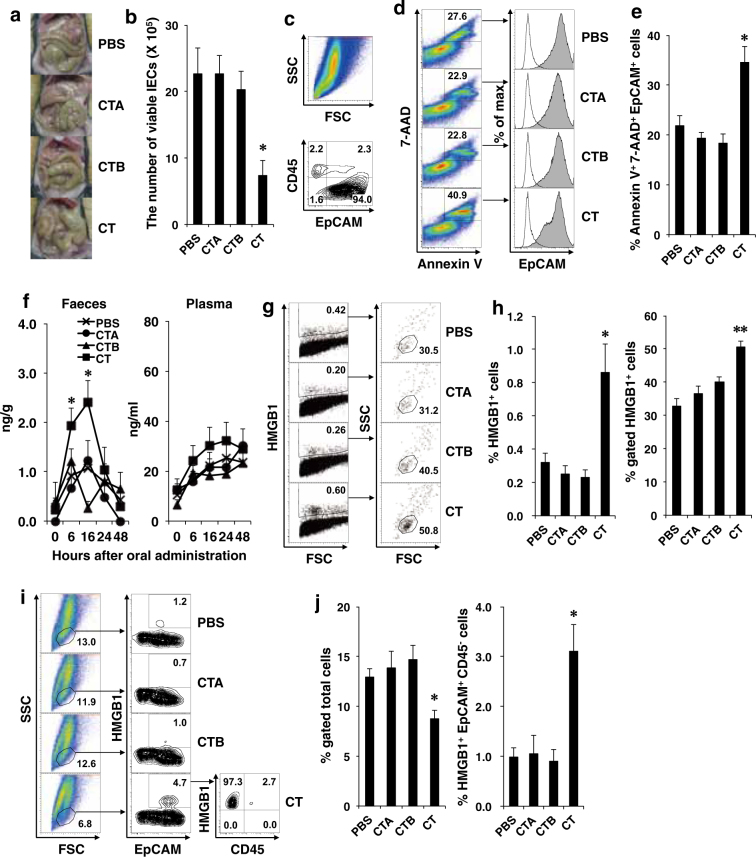
Oral administration of CT induces enhancement of intestinal cell death, cytoplasmic HMGB1 expression in IECs, and increased HMGB1 release. B6 mice were orally administered 10 μg of CTA, CTB, or intact CT. **a** Representative photographs of the intestine 16 h after oral administration. **b** IECs were isolated from the mice 16 h after oral administration and viable cells were counted using Trypan blue and microscopy. **c** IECs were stained with APC-labeled anti-EpCAM Ab and FITC-labeled anti-CD45 Ab. **d**, **e** IECs were stained with PE-labeled anti-EpCAM Ab and the APC Annexin V/7-AAD staining kit. **d** Annexin V^+^ 7-AAD^+^ cells were identified as dying cells and EpCAM expression was analyzed. **e** Data (%) for Annexin V^+^ 7-AAD^+^ EpCAM^+^ cells as described in (**e**) are shown as the mean + SE in three mice. The asterisk (*) indicates a statistically significant difference (*P* < 0.05) between the mice group treated with PBS ( × ), CTA (●), or CTB (▲); Welch’s *t*-test. **f** Feces and blood were collected 0, 6, 16, 24, and 48 h after the oral administration, and HMGB1 levels in fecal extracts and plasma were measured by ELISA assay. The results are shown as the mean + SE in four to seven mice per group. The asterisk (*) indicates statistically significant differences (*P* < 0.05) between the mouse groups treated with PBS ( × ) at 6 and 16 h after oral administration; Welch’s *t*-test. **g**–**j** IECs were isolated from mice 16 h after oral administration, stained with APC-labeled anti-EpCAM Ab and FITC-labeled anti-CD45 Ab, and then subjected to cytoplasmic staining with PE-labeled anti-HMGB1 Ab. **g** Cytoplasmic HMGB1^+^ cells were gated and their scatter properties (FSC/SSC) were analyzed. **h** Data (%) for cytoplasmic HMGB1^+^ cells among the total cells and HMGB1^+^ cells gated with the HMGB1^+^ cell-rich scatter gate as described in (**h**) are shown as the mean + SE in four to five mice. The single asterisk (*) and double asterisk (**) indicate statistically significant differences (*P* < 0.05 and *P* < 0.005, respectively) between the mouse groups treated with PBS ( × ), CTA (●), or CTB (▲); Welch’s *t*-test. **i** The HMGB1^+^ cell-rich population as described in (**h**) was gated and the expression of EpCAM and CD45 was analyzed. **j** Data (%) for the total cells gated with the HMGB1^+^ cell-rich scatter gate and cytoplasmic HMGB1^+^ EpCAM^+^ CD45^−^ cells as described in (**j**) are shown as the mean + SE in four to five mice. The asterisk (*) indicates a statistically significant difference (*P* < 0.05) between the mouse groups treated with PBS ( × ), CTA (●), or CTB (▲); Welch’s *t*-test

Oral administration of intact CT caused diarrhea and macroscopic erosion in the gut 6–24 h after administration, whereas oral CTA or CTB did not cause these morbid alterations macroscopically in the gut (Fig. 5a). Therefore, we examined whether oral administration of intact CT enhanced intestinal cell death and the release of HMGB1, which is a DAMP molecule, from the damaged or dead cells. The number of viable IECs was significantly reduced at 16 h after oral CT administration compared with oral administration of PBS, the CTA subunit, or the CTB subunit (Fig. 5b). The purity of the epithelial cell adhesion molecule (EpCAM)^+^CD45^−^ IEC population was consistently above 90% (Fig. 5c). The rate of dying Annexin V^+^ 7-AAD^+^ cells was significantly increased in EpCAM^+^ IECs at 16 h after oral administration of intact CT compared with other treatments (Fig. 5d,e), and the plots showed wide-ranging cell populations in forward scatter (FSC) and side scatter (SSC) parameters (Fig. S3). Next, we observed significant increases in the fecal HMGB1 levels at 6 and 16 h after oral CT administration compared with PBS treatment, although plasma HMGB1 levels did not significantly increase after the same oral administration (Fig. 5f). Cytoplasmic HMGB1^+^ IECs were significantly increased by oral CT administration compared with the other treatments, and these cells appeared to be a specific cell population in the FSC and SSC parameters (Fig. 5g,h). When we gated the cytoplasmic HMGB1^+^ cell population in the FSC/SSC of the total IECs, we observed a significant decrease in the gated cells and increase in the cytoplasmic HMGB1^+^EpCAM^+^CD45^−^IECs in response to oral CT administration (Fig. 5i,j).

Taken together, these results suggest that oral CT administration may induce damage and death of EpCAM^+^ IECs, cytoplasmic HMGB1 expression in the IEC population, and HMGB1 release in feces.

### HMGB1 enhances CD80 and CD86 expression on CD11c^+^ DCs in vitro

**Fig. 6:**
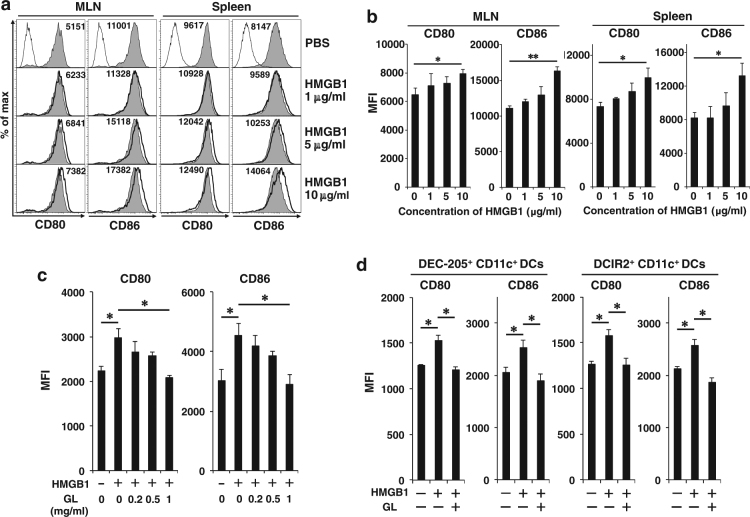
HMGB1 enhances CD80 and CD86 expression on DCs, including DEC-205^+^ DCs, in vitro. **a** MLN cells and splenic cells were isolated from B6 mice and treated with various concentration of HMGB1 (0, 1, 5, or 10 μg/ml) for 16 h. The cells were double-stained with FITC-labeled anti-CD11c Ab and APC-labeled anti-CD80, anti-CD86 (gray fill for HMGB1 0 μg/ml and bold solid line for 1, 5, and 10 μg/ml), or control Ab (solid line). CD11c^+^ cells were gated and the expression of CD80 or CD86 was analyzed. Each value represents the mean fluorescence intensity (MFI). **b** MFI data as described in (**a**) are shown as the mean + SE in four to five mice. *P < 0.05, **P < 0.0005; Welch’s *t*-test. **c** MLN cells were isolated from B6 mice and treated with HMGB1 (10 μg/ml) in the presence of various concentration of GL (0, 0.2, 0.5, 1 mg/ml) for 16 h. The cells were double-stained and analyzed by flow cytometry as described in (**a**). MFI data are shown as the mean + SE in three mice. *P < 0.05; Welch’s *t*-test. **d** MLN cells were isolated from B6 mice and treated with HMGB1 (10 μg/ml) in the presence or absence of GL (1 mg/ml) for 16 h. The cells were triple-stained with FITC-labeled anti-CD11c Ab, PE-labeled anti-DEC-205 or anti-DCIR2 Ab, and APC-labeled anti-CD80, anti-CD86, or subtype control Ab. DEC-205^+^CD11c^+^ cells and DCIR2^+^CD11c^+^ cells were gated and the expression of CD80 or CD86 was analyzed. Data of MFI are shown as the mean + SE in three mice. *P < 0.05; Welch’s *t*-test

Next, we examined whether HMGB1 could upregulate the expression of CD80 and CD86 on DCs in vitro. HMGB1 dose-dependently enhanced the expression of CD80 and CD86 on CD11c^+^ DCs from both MLNs and the spleen, and addition of 10 μg/ml HMGB1 significantly enhanced this expression (Fig. 6a,b); however, the direct addition of CT (10–50 μg/ml) did not activate DCs in vitro (Fig. S4). Glycyrrhizin (GL), an inhibitor of HMGB1, directly binds to HMGB1 and inhibits its activities^29^. GL dose-dependently inhibited CD80 and CD86 expression on MLN CD11c^+^ DCs that was enhanced by HMGB1, and significant inhibition was observed by addition of 1 mg/ml GL in vitro (Fig. 6c). HMGB1 significantly enhanced co-stimulatory molecule expression on both DEC-205^+^CD11c^+^ and DCIR2^+^CD11c^+^ DCs, and GL markedly inhibited it in vitro (Fig. 6d).

These data clearly show that HMGB1 activates DCs, including DEC-205^+^ DCs and DCIR2^+^ DCs, and the DC activation was strongly inhibited by addition of GL, an HMGB1 inhibitor, in vitro.

### HMGB1 released through oral CT administration contributes to the activation of intestinal DCs and cross-priming of CTLs

**Fig. 7:**
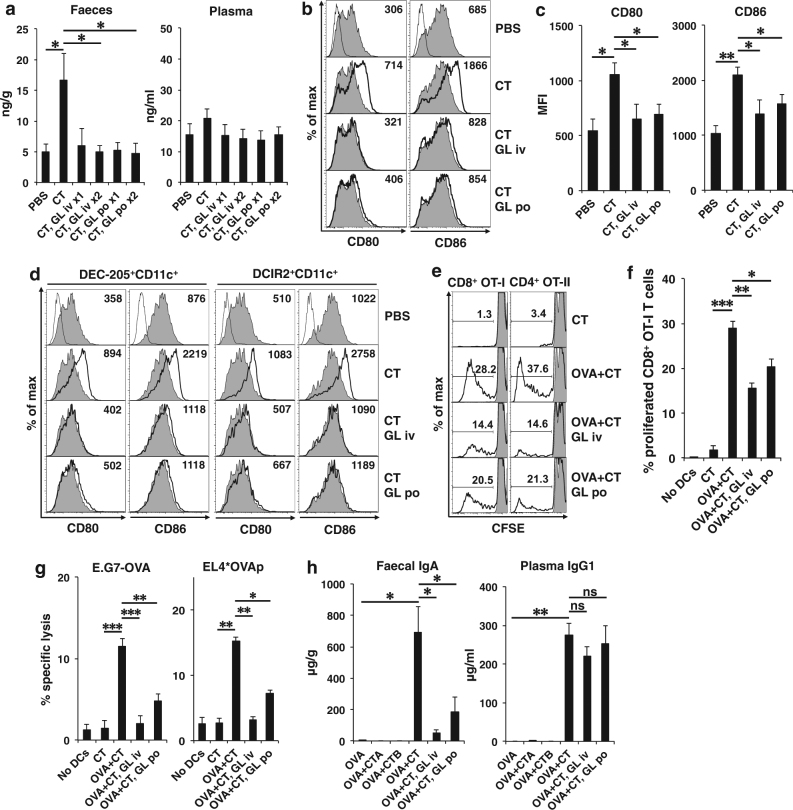
HMGB1 released through oral CT administration contributes to activation of intestinal DCs and cross-priming of CTLs in vivo. **a** B6 mice were orally administered PBS or 10 μg of CT and simultaneously treated with i.v. injection or oral administration of 500 μg of GL. Six hours later, some mice were re-treated with GL in the same manner. Feces and blood were collected 16 h after oral administration of CT and GL treatment, and HMGB1 levels in the fecal extracts and plasma were measured by ELISA assay. The results are shown as the mean + SE in four to five mice per group. *P < 0.05; Welch’s *t*-test. **b** B6 mice were orally administered PBS or 10 μg of CT and treated with i.v. injection or oral administration of 500 μg of GL at the same time and 6 h after CT administration. MLN cells were isolated from the mice 24 h after CT administration and double-stained with FITC-labeled anti-CD11c Ab and APC-labeled anti-CD80, anti-CD86 (gray fill for PBS-treated mice and bold solid line for mice treated with CT and GL), or subtype control Ab (solid line). CD11c^+^ cells were gated and the expression of CD80 or CD86 was analyzed. Each value represents the MFI of CD80 or CD86. **c** MFI data as described in (**b**) are shown as the mean + SE in four to six mice. *P < 0.05, **P < 0.001; Welch’s *t*-test. **d** MLN cells were isolated from mice treated as described in (**b**) and triple-stained with FITC-labeled anti-CD11c Ab, PE-labeled anti-DEC-205 or anti-DCIR2 Ab, and APC-labeled anti-CD80, anti-CD86, or subtype control Ab. DEC-205^+^CD11c^+^ cells and DCIR2^+^CD11c^+^ cells were gated and the expression of CD80 or CD86 was analyzed. The results are representative of two independent experiments. **e** B6 mice were orally administered 10 μg of CT alone or plus 10 mg of OVA and treated with i.v. injection or oral administration of 500 μg of GL at the same time and 6 h after the CT administration. MLN cells were isolated from mice 24 h after CT administration and CD11c^+^ DCs were purified from MLN cells using the MACS system and co-cultured with CFSE-labeled OT-I or OT-II T cells for 4 days. The cultured OT-I or OT-II T cells were stained with APC-labeled anti-CD8 Ab or anti-CD4 Ab, respectively. CD8^+^ OT-I or CD4^+^ OT-II T cells were gated and the CFSE dilution profiles were analyzed by flow cytometry. **f** Data for the proliferation of CD8^+^ OT-I T cells (%) as described in (**e**) are shown as the mean + SE in three mice. *P < 0.05, **P < 0.005, ***P < 0.001; Welch’s *t*-test. **g** The cultured OT-I T cells as described in (**e**) were re-stimulated by plate-bound anti-CD3 Ab for 16 h and OVA-specific cytotoxic activity of the re-stimulated OT-I T cells was assessed. The E:T ratio is 10:1 and the results are shown as the mean + SE in triplicate of pooled cells from two mice. *P < 0.01, **P < 0.005, ***P < 0.0005; Welch’s *t*-test. **h** B6 mice were orally administered 100 mg of OVA alone or plus 10 μg of CTA subunit, CTB subunit, or CT every week. Some of the mice administered with OVA plus CT were treated by i.v. injection or oral administration of 500 μg of GL concurrently with, and 6 h after, the administration of OVA plus CT every week. Feces and blood were collected 3 weeks after the first oral administration and levels of anti-OVA IgA in the fecal extracts and anti-OVA IgG1 in the plasma were measured by ELISA assay. The results are shown as the mean + SE (four to five mice per group). *P < 0.05, **P < 0.001, not significant (ns); Welch’s *t*-test

Finally, we examined whether HMGB1 released by the oral administration of CT could mediate DC activation and cross-priming of CD8^+^ CTLs in vivo. The terminal half-life of GL in plasma is 3–5 h after intravenous (i.v.) administration^30^. However, after oral administration, GL is not detected in the plasma and 1.1–2.5% of the dose is found in urine, suggesting that GL is partly absorbed in the intact form from the gastrointestinal tract^30^. Two i.v. or oral treatment of B6 mice with the HMGB1 inhibitor GL concurrently with, and 6 h after, oral CT administration significantly suppressed the increased HMGB1 levels in fecal extracts, although only one i.v. or oral treatment by GL at the time of CT administration tended to suppress HMGB1 levels (Fig. 7a). Therefore, we decided to treat B6 mice twice with GL as the HMGB1 inhibitor. Intravenous or oral treatment with GL significantly suppressed the enhancement of CD80 and CD86 expression on MLN CD11c^+^ DCs (Fig. 7b,c), including both DEC-205^+^CD11c^+^ and DCIR2^+^CD11c^+^ DCs (Fig. 7d), by oral CT administration. Intravenous or oral treatment with GL also suppressed MHC class I expression on MLN CD11c^+^CD11b^−^DEC205^+^ DCs that had been enhanced by oral CT administration (Fig. S5). These results suggested that HMGB1 released after oral CT administration mediated the activation of both DEC-205^+^CD11c^+^ DCs and DCIR2^+^CD11c^+^ DCs in MLNs. We further assessed whether the inhibition of HMGB1 release also suppressed in vivo antigen presentation of DCs. Accordingly, i.v. or oral treatment with GL significantly suppressed the ability of DCs to induce OVA-specific CD8^+^ OT-I or CD4^+^ OT-II T-cell proliferation and OVA-specific CTL activity of OT-I T cells by oral administration of OVA plus CT (Fig. 7e-g). Oral administration of OVA plus CT adjuvant remarkably induced production of anti-OVA fecal IgA and plasma IgG1 as previously reported^1^; however, Ig production was not observed by oral administration of OVA alone or plus the CTA or CTB subunit (Fig. 7h). Intravenous or oral treatment with the HMGB1 inhibitor, GL, significantly suppressed the fecal anti-OVA IgA levels that had been increased by oral administration of OVA plus CT, although the GL treatment did not suppress plasma anti-OVA IgG1 levels (Fig. 7h).

These results demonstrate that HMGB1 released from intestinal mucosa through oral CT administration contributes to the activation of mucosal DCs and induction of intestinal antigen-specific CD8^+^ CTLs and IgA.

## Discussion

We observed that CT could not directly enhance CD80 and CD86 expression on CD11c^+^ DCs in vitro (Fig. S4), in contrast to reports showing that CT upregulates the expression of MHC class II, B7.1 and B7.2 molecules, on DCs^31,32^. In our experimental system, we took care to exclude lipopolysaccharide contamination in the cultures. Thus, we hypothesized that the administered CT did not directly act on the intestinal DCs but could trigger the induction or release of DC activators from IECs.

In fact, we showed that intact CT strongly stimulated cell death of EpCAM^+^ IECs (Fig. 5). Severe damage to IECs induces the release of DAMP molecules, such as intracellular proteins or fragmented nucleic acid^33^. HMGB1 is normally located in the nucleus, but nuclear HMGB1 translocates to the cytosol following exposure to various stressors, such as starvation, rapamycin, or oxidative stress, and cytoplasmic HMGB1 promotes autophagy by binding to Beclin 1^34,35^. HMGB1 can be passively released from dead, dying, or damaged cells by necrosis^36^, apoptosis^37^, autophagy^35^, or necroptosis^38^, and is actively secreted by activated immune cells, such as DCs^39^ and macrophages^40^. Intact CT may trigger damage or death of IECs, the translocation of nuclear HMGB1 to the cytoplasm in damaged IECs, and HMGB1 release from these cells. However, it is unclear whether specific IECs are damaged and what type of cell death, e.g., necrosis, apoptosis, or necroptosis, occurs in IECs in response to CT administration.

Released extracellular HMGB1 acts as a DAMP, which is an alarmin signal, and the released DAMP molecules cause inflammation^16,17^. We showed that HMGB1 activated murine DCs and upregulated the expression of co-stimulatory molecules on DCs, such as DEC-205^+^ DCs (Fig. 6), as reported in human^18^ and rat DCs^19^. However, oral administration of either the CTA or CTB subunit did not enhance cell death of IECs, expression of cytoplasmic HMGB1, and HMGB1 release in the feces (Fig. 5). Therefore, oral administration of the CTA or CTB subunit could not activate MLN DCs in mice (Fig. 2). We found that HMGB1 mediated DC activation and cross-presentation of an antigen by oral CT administration (Fig. 7). TLR2 and 4, and the receptor for advanced glycation end products are receptors of extracellular HMGB1^41,18^ and we will determine which receptors are involved in intestinal DC activation in future studies. Other DAMPs, intracellular components such as S100 proteins or fragmented nucleic acids^33^, may be released from the damaged IECs and be involved in induction of immune responses.

Consistent with a previous report showing cross-presentation in DEC-205^+^ DCs^9^, CD11c-DTR mice depleted of CD11c^+^ DCs, including DEC-205^+^ DCs, failed to induce intestinal CTLs (Fig. 3), and DCs that had captured antigen through the DEC-205 receptor showed strong cross-presentation of the antigen in vitro (Fig. 4h). CD8^+^ DCs were reported to be involved in the cross-presentation of antigen^6^; however, we showed that not only CD8^+^ MLN DCs but also CD8^−^ MLN DCs could cross-present OVA antigen when mice were orally administered OVA plus CT (Fig. 4g). This phenomenon may be due to the expression of DEC-205 on both CD8^+^ MLN DCs and CD8^−^ MLN DCs (Fig. 1). Thus, DEC-205 expression on DCs may be critical for the functional cross-presentation of an antigen, but CD8 expression may not necessarily correspond to the cross-presentation ability. However, the possible involvement of CD11c^+^ macrophages in cross-presentation is still unclear^42^.

Thus, CD11c^+^ DCs isolated from the intestinal LP, MLNs, or spleen could cross-present antigen when antigen was delivered through the DEC-205 receptor in vitro (Fig. 4h). However, when mice were orally administered an antigen plus intact CT, activation and cross-presentation were not observed in splenic DCs, although LP DEC-205^+^ DCs and MLN DEC-205^+^ DCs were activated and contributed to antigen cross-presentation (Figs. 2 and 4). LP DEC-205^+^ DCs locally capture antigen and are activated by HMGB1 released from the IECs damaged by oral CT. They may subsequently migrate to the draining MLNs in a CCR7-dependent manner^43^, but they did not migrate to the spleen sufficiently to cross-present antigen after oral administration of an antigen plus CT (Fig. 4). Notably, expression of MHC class I and co-stimulatory molecules was upregulated on DEC-205^+^CD11c^+^CDb^−^ DCs in the MLNs, but not in the LP and spleen, by oral CT administration (Fig. 2, Fig. S5), suggesting that the DC subset that migrates to the MLNs may effectively contribute to cross-presentation of an antigen and cross-priming of CD8^+^ CTLs.

Mucosal IgA production was induced in mice by using intact CT adjuvant but not the CTA or CTB subunit and was significantly suppressed by treatment with HMGB1 inhibitor (Fig. 7), indicating that HMGB1 contributes to antigen presentation via MHC class II, CD4^+^ T-cell priming and plasma cell differentiation in the intestine. Some plasma cells may migrate to systemic lymphoid tissues and produce IgG. Systemic IgG production could not be suppressed by local inhibition of HMGB1 release (Fig. 7).

Our study identified an important role of HMGB1 released from IECs damaged by oral CT administration in the induction of antigen-specific CTLs and IgA in the intestine. Unfortunately, it is difficult to use intact CT as a vaccine adjuvant for clinical applications because of its toxicity. However, elucidation of the mechanisms underlying activation of intestinal DCs and successful CTL induction may contribute to the development of a novel vaccine strategy for cancer or infectious diseases. After binding of the CTB subunit to monosialoganglioside 1 on the cell surface, the CTA subunit enters IECs, leading to increased intracellular cAMP and diarrhea^11,12^, whereas the intracellular CTA subunit may trigger transfer of nuclear HMGB1 to the cytoplasm and its subsequent extracellular release. It may be worthwhile to prepare a novel CT adjuvant with the CTA subunit modified to inactivate the ADP-ribosyltransferase activity and to examine whether it can trigger release of HMGB1 from IECs without causing diarrhea.

## Materials and methods

### Mice

C57BL/6J (B6; H-2^b^) mice were obtained from CLEA Japan, Inc. (Tokyo, Japan). B6.FVB-Tg(Itgax-DTR/EGFP)57Lan/J Tg (CD11c-DTR)^27^, C57BL/6-Tg(TcraTcrb)1100Mjb/J Tg (OT-I)^44^, and B6.Cg-Tg(TcraTcrb)425Cbn/J Tg (OT-II) mice^45^ were obtained from The Jackson Laboratory (Bar Harbor, ME). The mice were maintained in micro-isolator cages under pathogen-free conditions and fed autoclaved laboratory chow and water. All animal experiments were performed according to the guidelines for the care and use of laboratory animals established by the National Institutes of Health (Bethesda, MD) and approved by the Review Board of Nippon Medical School (Tokyo, Japan).

### Isolation of DCs from LP, MLNs, and spleen

LP cells were also prepared using a previously described method^43,46^. The small intestine was removed from mice and the Peyer’s patches were cut away. The intestine was cut lengthwise, washed with PBS to remove fecal material, and cut into 1 cm segments. The gut segments were stirred in 50 ml of Hanks’ balanced salt solution (HBSS, Invitrogen, Carlsbad, CA) with 5 mM EDTA (Wako Pure Chemical Industries, Osaka, Japan), 10 µg/ml polymixin B (Sigma-Aldrich, St. Louis, MO), 100 U/ml penicillin, and 100 µg/ml streptomycin (Invitrogen) at 37 °C for 20 min, washed extensively with PBS, and minced with scissors. The minced gut was then stirred in 20 ml of HBSS with 10% fetal calf serum (FCS), 2 mg/ml collagenase D (Roche, Basel, Switzerland), and 200 µg/ml DNase I (Roche) at 37 °C for 60 min, and the collected cells were passed through a nylon mesh. The LP cells were suspended in 30% Percoll solution (GE Healthcare, Uppsala, Sweden). The solution was underlain with 60% Percoll and centrifuged at 950 × *g* for 20 min. The DC-rich fraction was collected from the 30/60% interface. The MLNs and spleens were aseptically removed from the mice and incubated in 1 mg/ml collagenase D at 37 °C for 45 min. A single cell suspension was underlain with 60% Percoll and centrifuged at 950 × *g* for 20 min. The DC-rich fraction was collected from the interface, washed, and counted. The DC-rich fraction from LP, MLN, or splenic cells was used for flow cytometry analysis or purification of CD11c^+^ DCs by the MACS (Miltenyi Biotec, Bergisch Gladbach, Germany) system. Pan-DCs were obtained from the DC-rich fraction by depletion of T, B, and natural killer cells, and then CD8^+^ DCs were purified from pan-DCs by CD8α MicroBeads using the MACS system. DCs unlabeled with CD8α MicroBeads were collected and used as CD8^−^ DCs.

### DC depletion in CD11c-DTR Tg mice

CD11c-DTR Tg and WT B6 mice were i.p. injected with 4 ng of DT (Wako Pure Chemical Industry) per gram of body weight.

### Oral immunization

OVA, chicken egg, grade V (Sigma-Aldrich) was dissolved in sterilized PBS. Mice were orally administered 10 or 100 mg of OVA alone or plus 10 µg of CTA subunit, CTB subunit, or CT (List Biological Lab., Campbell, CA) or 10 µg of CTA subunit, CTB subunit, or CT alone in 0.3 ml of PBS.

### Isolation of IELs and IECs

IELs and IECs were prepared using a previously described method^2,47^. The small intestine was obtained from mice, fecal materials were flushed from the lumen with HBSS, and connective tissues were carefully removed. The guts were inverted, cut into several segments and shaken at 200 r.p.m. in 45 ml of HBSS with 5% FCS, 100 U/ml penicillin, and 100 µg/ml streptomycin at 37 °C for 45 min. For isolation of IELs, collected cells from the intestinal epithelium were passed through a glass wool column to remove tissue debris. Subsequently, the cells were suspended in 30% Percoll and centrifuged at 600 g for 20 min. The cells at the bottom were resuspended in 44% Percoll, underlain with 70% Percoll, and centrifuged at 600 × *g* for 20 min. IELs were collected from the 44/70% interface. For isolation of IECs, the cells released upon shaking were resuspended in 25% Percoll, underlain with 40% Percoll, and centrifuged at 600 × *g* for 10 min. IECs were collected from the 25/40% interface. The purity of the IEC population was assessed by fluorescence-activated cell sorting analysis for the EpCAM using phycoerythrin (PE)- or allophycocyanin (APC)-labeled anti-mouse EpCAM (CD326) Ab (BioLegend, San Diego, CA) and was consistently above 90%.

### Flow cytometry analysis

Cells were pre-incubated with anti-CD16/32 Ab (24G2, prepared from hybridoma supernatant) to block nonspecific FcR-mediated Ab staining. To stain DCs, fluorescently (fluorescein isothiocyanate (FITC), PE, or APC) labeled anti-mouse CD11c (N418), CD11b (M1/70), CD8α (53–6.7), DEC-205 (NLDC-145), DCIR2 (33D1), CD40 (1C10 or 3/23), CD80 (16–10A1), CD86 (GL-1), MHC class I (H-2K^b^, AF6-88.5), or MHC class II (I-A/I-E, M5/114.15.2), and each subtype control Ab were obtained from BioLegend, and PE-labeled anti-mouse CD103 (M290) Ab was purchased from BD Biosciences (San Jose, CA). Cells were double- or triple-stained with FITC, PE, or APC-labeled Ab. For detection of OVA-specific CTLs, cells were double-stained with PE-labeled H-2K^b^/OVA tetramer-SIINFEKL (Medical & Biological Laboratories, Nagoya, Japan) and APC-labeled anti-mouse CD8β (Ly-3) Ab (BioLegend). IECs were stained with PE-labeled anti-mouse EpCAM (CD326, G8.8) Ab and cell death in IECs was evaluated using an APC Annexin V/7-AAD staining kit (BioLegend). For detection of cytoplasmic HMGB1, IECs were stained with APC-labeled anti-mouse EpCAM Ab and FITC-labeled anti-mouse CD45 (30-F11) Ab, and then intracellular staining with PE-labeled anti-HMGB1 (3E8) Ab (BioLegend) was performed using the BD Cytofix/Cytoperm Kit (BD Biosciences). All data were acquired using a FACSCant II flow cytometer (BD Biosciences) and analyzed using FlowJo software (Tree Star, Ashland, OR).

### CTL assay

Cytolytic activity was measured using a standard ^51^Cr-release assay as previously described^2,48^. OVA-peptide (257–264, SIINFEKL)-pulsed EL4 cells (H-2^b^) and E.G7-OVA cells, which are OVA gene-transfected EL4 cells^49,50^ were used as target cells. EL4 and E.G7-OVA cells were obtained from the American Type Culture Collection (Manassas, VA). Effector cells were incubated with 3 × 10^3 51^Cr-labeled targets at 37 °C for 4 h in 200 µl of RPMI 1640 medium containing 10% FCS in round-bottomed 96-well cell culture plates. After incubation, 100 µl of cell-free supernatants was collected to measure radioactivity with a Packard Auto-Gamma 5650 counter (Hewlett-Packard, Tokyo, Japan). Maximum release was determined in the supernatant of cells that had been lysed by addition of 5% Triton X-100 and spontaneous release was determined from target cells incubated without added effector cells. The percent specific lysis was calculated as follows: 100 × (experimental release − spontaneous release)/(maximum release − spontaneous release).

### Proliferation of CSFE-labeled OT-I or OT-II T cells by co-culture with DCs

The spleens and MLNs were removed from OT-I or OT-II mice. A single cell suspension was prepared from spleens and MLNs, and red blood cells were depleted by cell lysis. OT-I or OT-II T cells were isolated using a nylon wool column and labeled with 2.5 μM CFSE (Invitrogen) for 10 min at room temperature in the dark. Two to 3 × 10^6^ CFSE-labeled OT-I T cells were co-cultured with a 1–4 × 10^5^ MACS-purified DC population in 0.5 ml of complete T-cell medium (CTM)^2^ composed of RPMI 1640 medium supplemented with 2 mM l-glutamine, 1 mM sodium pyruvate, 0.1 mM non-essential amino acids, MEM vitamins, 1 mM HEPES, 100 U/ml penicillin, 100 μg/ml streptomycin, 50 μM 2-ME, and heat-inactivated 10% FCS in 48-well culture plates at 37 °C in 5% CO_2_ for 4 days. The cultured OT-I or OT-II T cells were treated with anti-CD16/32 Ab for blocking and stained with APC-labeled anti-mouse CD8α (53–6.7) or CD4 (RM4-5) Ab (BioLegend), respectively. The CFSE dilution profiles of CD8^+^ OT-I or CD4^+^ OT-II T cells were analyzed by flow cytometry.

### Targeting OVA to DCs via the DEC-205 uptake receptor

OVA targeting to CD11c^+^ DCs via DEC-205 or DCIR2 receptor was performed using an OVA antigen delivery module set (Miltenyi Biotec). CD11c^+^ DCs were purified by the MACS system and labeled using biotinylated anti-DEC-205 Ab, anti-DCIR2 Ab, or subtype control Ab. Subsequently, the cells were loaded with anti-biotin Ab conjugated to OVA antigens.

### Restimulation of OT-I T cells by anti-CD3 Ab

The cultured OT-I T cells were re-stimulated using a previously described method^51^. OT-I T cells were recovered from co-cultures with DCs on day 4. The 10^5^ T cells were re-stimulated by plate-bound anti-CD3 Ab (145.2C11, 10 μg/ml; Miltenyi Biotec) in 200 μl of CTM in flat-bottomed 96-well plates at 37 °C in 5% CO_2_ for 16 h. The culture supernatant and T cells were collected for IFN-γ measurement and the CTL assay, respectively. IFN-γ release into the supernatant was measured using an IFN-γ enzyme-linked immunosorbent assay (ELISA) kit (BD Biosciences).

### Measurement of levels of HMGB1, anti-OVA IgA, and IgG1 by ELISA assay

Peripheral plasma and fecal samples were collected from mice as previously described^1^. Peripheral blood was collected from anaesthetized mice using heparinized capillary tubes and centrifuged at 6000 r.p.m. and 4 °C for 10 min. Plasma was collected and stored frozen at − 80 °C until use. Fresh feces were collected and weighed, followed by addition of PBS (200 mg/ml). The feces in PBS were homogenized by continuous shaking for 10 min with a vortex and centrifugation at 10,000 r.p.m. at 4 °C for 10 min. Supernatants were collected and stored frozen at − 80 °C until use. The concentrations of HMGB1 were measured in plasma and fecal samples using the HMGB1 ELISA kit II (Shino-Test Corporation, Tokyo, Japan). Anti-OVA IgA and IgG1 levels were determined by ELISA as described previously^1^. Flat-bottomed 96-well microtitre plates were coated with 100 µL of 1 mg/ml OVA (Sigma-Aldrich) in carbonate buffer at 4 °C for 16 h. After washing with PBS containing 0.05% Tween-20, the wells were blocked with 1% bovine serum albumin in PBS 37 °C for 1 h. After washing, diluted plasma or fecal extract samples were added to the wells in duplicate and incubated at 37 °C for 1 h. After washing, biotinylated anti-mouse IgA or IgG1 (BD Biosciences) were added to the wells and incubated at 37 °C for 1 h. After washing, horseradish peroxidase-conjugated streptavidin (Caltag Laboratory, Burlingame, CA) was then added and incubated at 37 °C for 30 min. Enzymatic detection was performed with TMB (3,3′,5,5′-tetramethylbenzidine) soluble reagent (ScyTek Laboratories, Logan, UT) and terminated by the addition of 2 N HCl followed by absorbance measurement at 450 nm. For a standard curve, part of the assay plate was coated with various quantities of purified mouse IgA (ICN/Cappel, Aurora, OH) or IgG1 (BD Biosciences). After blocking, biotinylated anti-mouse IgA or IgG1 was added to the wells and the reaction was performed in the same manner as the sample wells.

### In vitro treatment of DCs with HMGB1 and its inhibitor GL

The DC-rich fraction was prepared from MLNs or spleens using collagenase D and 60% Percoll as described above. Six to 10 × 10^6^ cells were cultured in the presence of 1, 5, or 10 μg/ml bovine HMGB1 (Chondrex, Redmond, WA) or CT with or without GL (Wako Pure Chemical Industries) in 0.5 ml of CTM in 48-well culture plates at 37 °C in 5% CO_2_ for 16 h.

### In vivo CT administration and GL treatment

Mice were orally administered 10 µg of CT alone or plus 100 mg of OVA and simultaneously treated with i.v. injection or oral administration of 500 μg of GL. Six hours later, the mice were re-treated with GL in the same manner.

### Statistical analysis

All data are represented as the mean ± SE. Statistical analysis was carried out using the *t*-test assuming unequal variances (Welch’s *t*-test). *P*-value ≤ 0.05 was considered significant.

## Electronic supplementary material

Supplemental Figures S1-5

Supplementary figure legends
