# Supplementary figures and images for: HMGB1 released from intestinal epithelia damaged by cholera toxin adjuvant contributes to activation of mucosal dendritic cells and induction of intestinal cytotoxic T lymphocytes and IgA
Source: Cell Death Dis. 2018 May 24;9(6):631. doi: 10.1038/s41419-018-0665-z (PMC5967345; doi:10.1038/s41419-018-0665-z)

Fig. S1

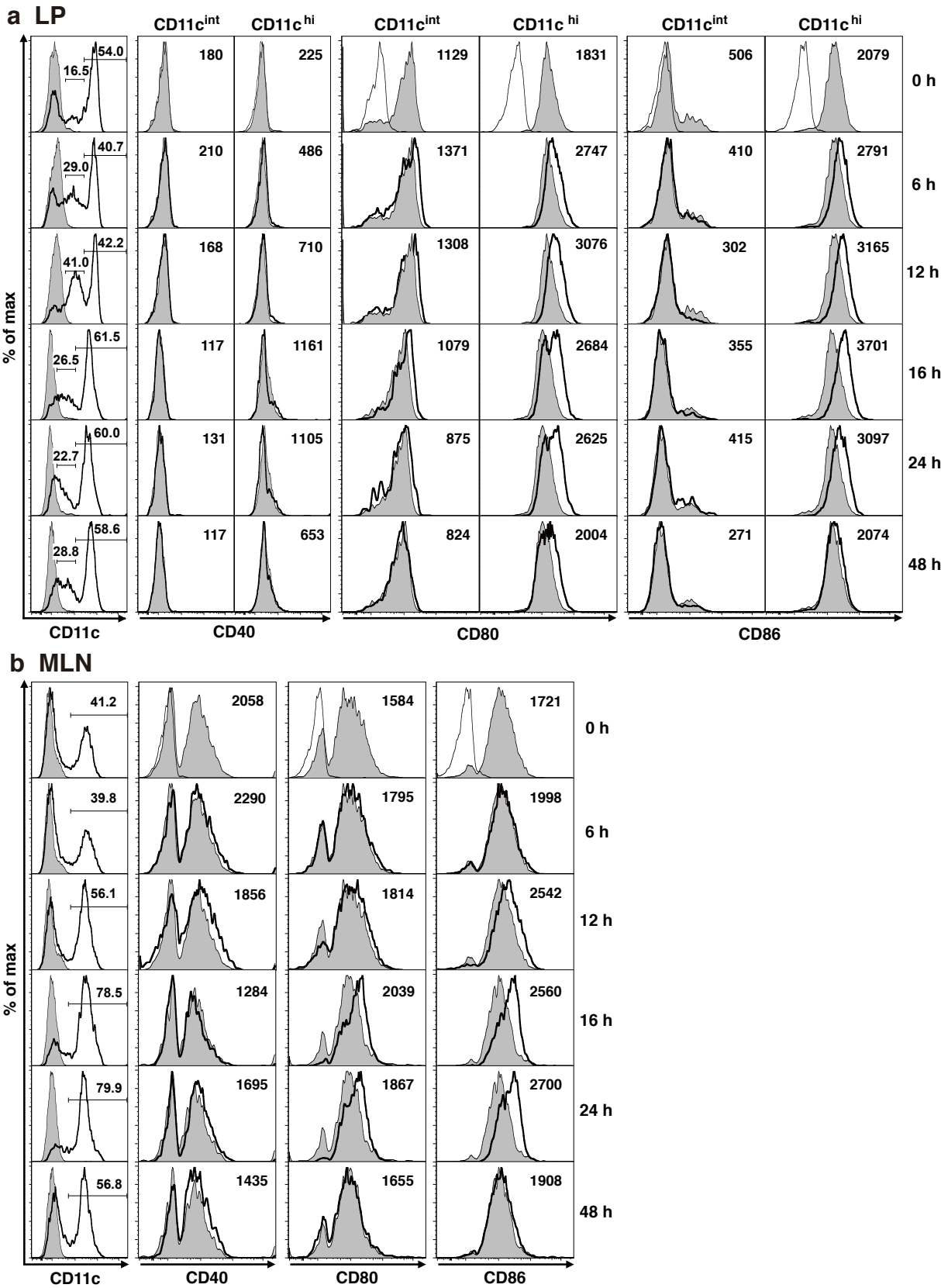

Fig. S2

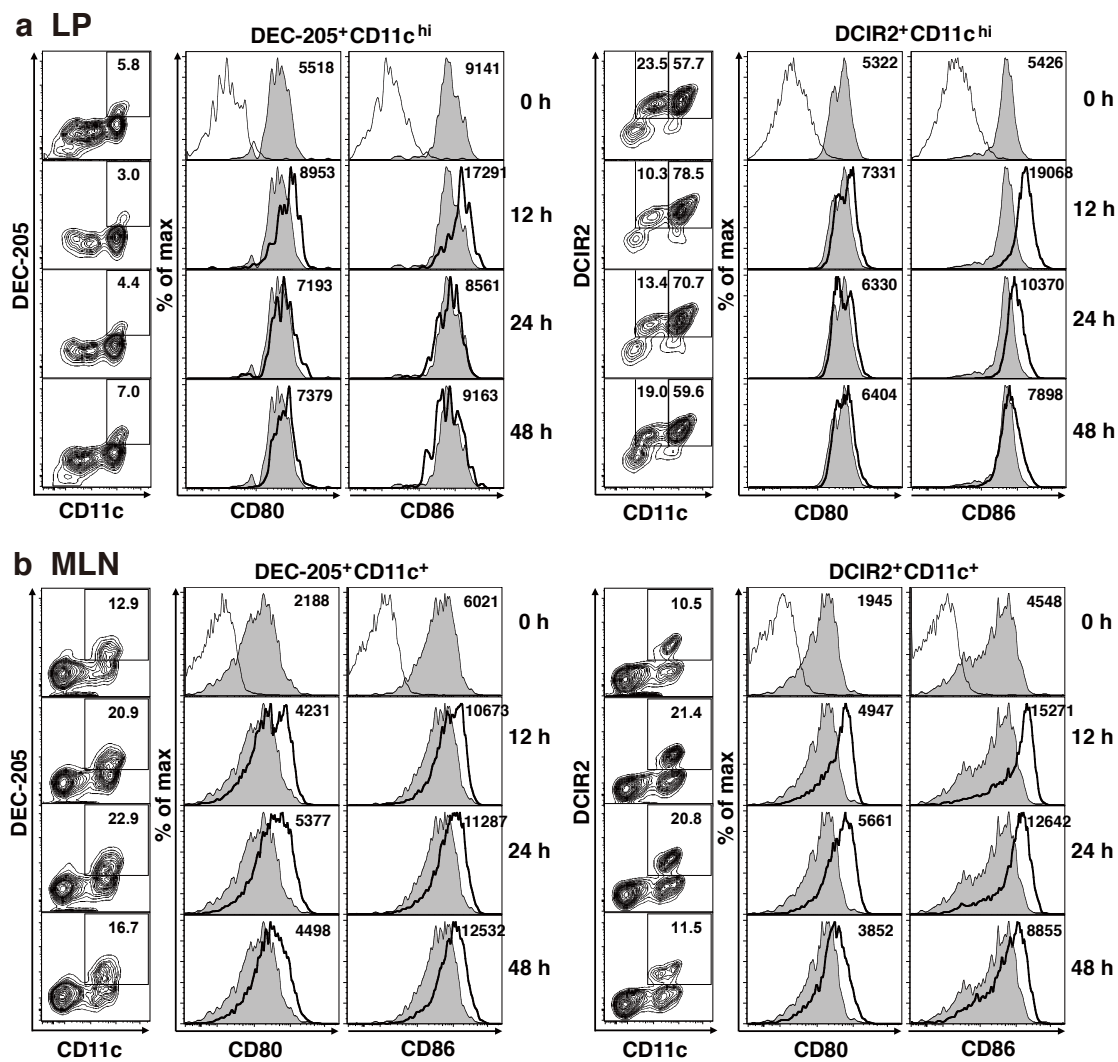

Fig. S3

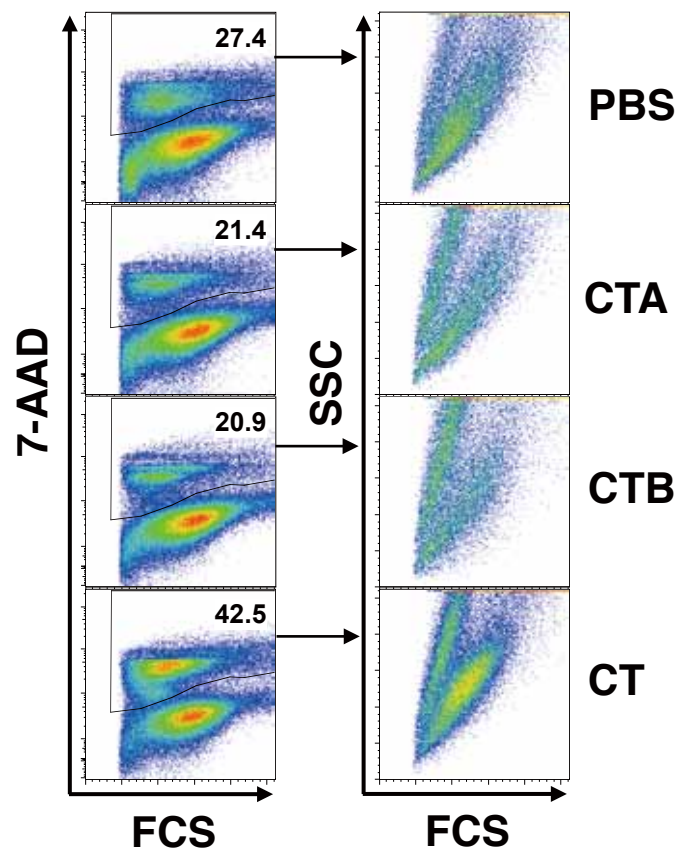

Fig. S4

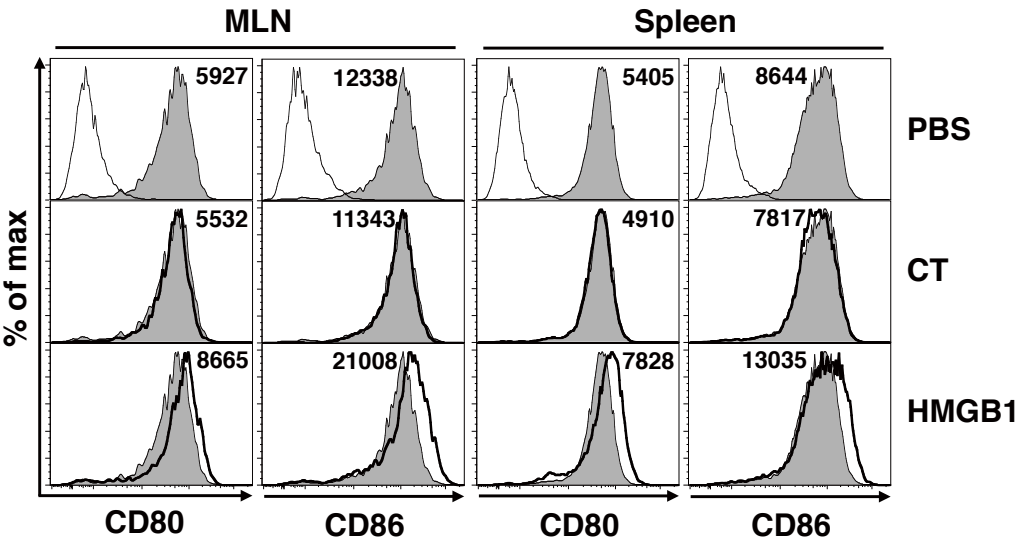

Fig. S5

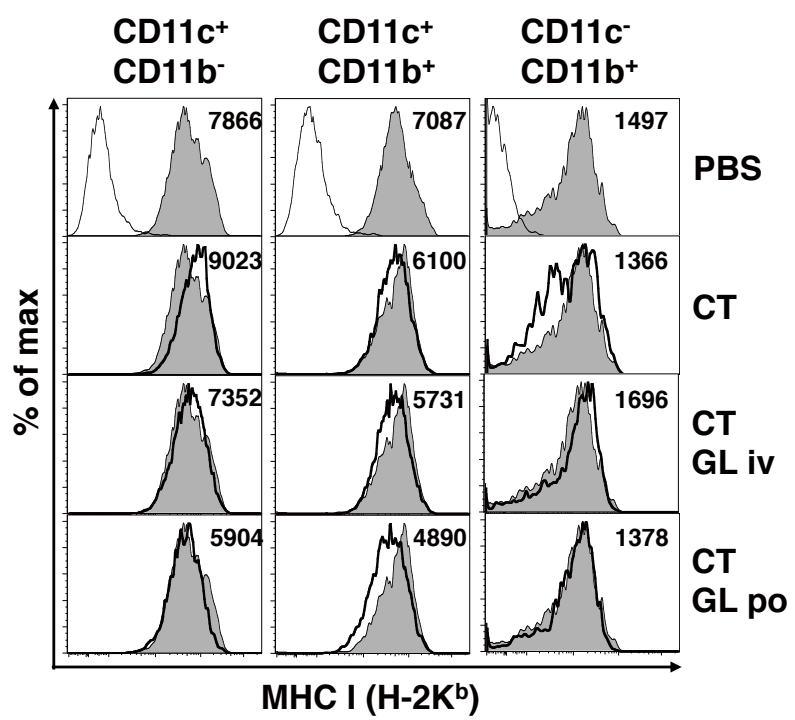

Supplement: Supplementary file 1 — Supplemental Figures S1-5 [file 41419_2018_665_MOESM1_ESM.pdf]
