# Supplementary material for: HMGB1 released from intestinal epithelia damaged by cholera toxin adjuvant contributes to activation of mucosal dendritic cells and induction of intestinal cytotoxic T lymphocytes and IgA
Source: Cell Death Dis. 2018 May 24;9(6):631. doi: 10.1038/s41419-018-0665-z (PMC5967345; doi:10.1038/s41419-018-0665-z)
Supplement: Supplementary file 2 — Supplementary figure legends [file 41419_2018_665_MOESM2_ESM.doc]

## Supplementary figure legends

**Fig. S1:** Enhancement of CD80 and CD86 expression on intestinal CD11c^+^ DCs after oral administration of CT. **a, b** B6 mice were orally administered 10 μg of CT. LP cells (**a**) and MLN cells **b** were isolated from the mice 0, 6, 12, 16, 24, and 48 h after oral administration and double-stained with FITC-labeled anti-CD11c (left panel, bold solid line) or subtype control Ab (left panel, gray fill) and PE-labeled anti-CD40, anti-CD80, anti-CD86 (gray fill for 0 h and bold solid line for 6, 12, 16, 24, and 48 h), or control Ab (solid line). CD11c^high^ and CD11c^low^ LP (**a**) or CD11c^+^ MLN (**b**) cells were gated (each left panel), and the expression of CD40, CD80, or CD86 was analyzed. Each value represents the percentage of cells expressing CD11c (each left panel) and the mean fluorescence intensity (MFI) of CD40, CD80, or CD86. Data are representative of two independent experiments.

**Fig. S2:** Enhancement of CD80 and CD86 expression on intestinal DEC-205^+^ DCs and DCIR2^+^ DCs after oral administration of CT. **a, b** B6 mice were orally administered 10 μg of CT. LP (**a**) and MLN (**b**) cells were isolated from the mice 0, 12, 24, and 48 h after oral administration and triple-stained with FITC-labeled anti-CD11c Ab (each left panel), PE-labeled anti-DEC-205 or anti-DCIR2 Ab, and APC-labeled anti-CD80, anti-CD86 (gray fills for 0 h and bold solid line for 12, 24, and 48 h), or subtype control Ab (solid line). DEC-205^+^CD11c^+^ LP, DCIR2^+^CD11c^high^ LP*,* DEC-205^+^CD11c^+^ MLN, LP, and DCIR2^+^CD11c^+^ MLN cells were gated (each left panel), and the expression of CD80 or CD86 was analyzed. Each value represents the percentage of gated cells (each left panel) and the MFI of CD80 or CD86. Data are representative of two independent experiments.

**Fig. S3:** The plots of 7-AAD^+^ cells show wide-ranging cell population in the FSC/SSC scatter. IECs were stained with PE-labeled anti-EpCAM Ab and the APC Annexin V/7-AAD staining kit. 7-AAD^+^ cells were gated, an their scatter properties (FSC/SSC) were analyzed.

**Fig. S4:** CT does not enhance CD80 and CD86 expression on MLN DCs and splenic DCs *in vitro*. MLN and splenic cells were isolated from B6 mice and treated with CT or HMGB1 (10 μg/ml) for 16 h. The cells were double-stained with FITC-labeled anti-CD11c Ab and APC-labeled anti-CD80, anti-CD86 (gray fill for HMGB1 0 μg/ml and bold solid line for 1, 5, and 10 μg/ml), or control Ab (solid line). CD11c^+^ cells were gated, and the expression of CD80 or CD86 was analyzed. Each value represents MFI. Data are representative of two independent experiments.

**Fig. S5:** I.v. or oral treatment of GL suppresses the MHC class I expression on MLN CD11c^+^CD11b^−^ DCs that is enhanced by oral CT administration. B6 mice were orally administered 10 μg of CT and treated with i.v. injection or oral administration of 500 μg of GL concurrently with, and 6 h after, CT administration. MLN cells were isolated from the mice 24 h after oral administration and triple-stained with FITC-labelled anti-CD11b, APC-labelled anti-CD11c and PE-labelled anti-MHC class I (H-2K^b^) Abs (gray fill for PBS-treated mice and bold solid line for mice treated with CT and GL) or with subtype control Ab (solid line). CD11c^+^CD11b^−^, CD11c^+^CD11b^+^ and CD11c^−^CD11b^+^ cells were gated, and the expression of MHC class I was analyzed. Each value represents MFI. Data are representative of two independent experiments.
